# Stories for Change: The impact of Public Narrative on the co‐production process

**DOI:** 10.1111/hex.13718

**Published:** 2023-01-27

**Authors:** Sophie Moniz, Amelia Karia, Ahmad Firas Khalid, Cecilia Vindrola‐Padros

**Affiliations:** ^1^ Department of Targeted Intervention, Rapid Research Evaluation and Appraisal Lab (RREAL) UCL London UK; ^2^ Centre for Implementation Research, Canadian Institutes of Health Research Health System Impact Fellowship, Centre for Implementation Research Hospital Research Institute Ottawa Ontario Canada; ^3^ Health in Emerrgencies The Canadian Red Cross Ottawa Ontario Canada

**Keywords:** co‐design, co‐production, healthcare, maternity services, Public Narrative

## Abstract

**Introduction:**

Involving service users in health service design and delivery is considered important to improve the quality of healthcare because it ensures that the delivery of healthcare is adapted to the needs of the users. Co‐production is a process used to involve service users, but multiple papers have highlighted the need for the mechanisms and values guiding co‐production to be more clearly stated. The aim of this paper was to evaluate the mechanisms and values that guided the co‐production approach of the Stories for Change project, which used Public Narrative as part of the co‐design process to create change in National Health Service maternity services.

**Methods:**

This study was conducted using a rapid feedback evaluation approach. Semistructured interviews (*n* = 16) were the main source of data, six of which were maternity service users, with observations (5 h) and documentary analysis also carried out in parallel. RREAL sheets were used for data analysis to organize data based on key topics of interest.

**Results:**

This study identified three broad mechanisms and values underpinning the co‐production approach: creating an open and safe space to share ideas, learning how to tell stories using Public Narrative and having service providers who play a key role in strengthening the health system listen to stories compelling them to action. This study identified the main areas for improvement of the Stories for Change project related to recruitment, the inclusion of participants, the co‐design process, the Skills Session and the Learning Event.

**Conclusion:**

Our study provided a deeper understanding of the co‐production approach that addresses the need to uncover the mechanism and values underlying co‐production and co‐design approaches. This study expands on the literature pertaining to the influence of storytelling in creating meaningful change in health care. We propose a co‐design methodology that uses Public Narrative as a model for service user engagement to help inform future healthcare development processes.

**Patient or Public Contribution:**

The experiences and perceptions of maternity service users and health professionals informed this evaluation. The project organizers were involved in the manuscript preparation stage by providing feedback, and service users wrote a commentary on the project from the lived experience perspective.

## 1 INTRODUCTION

In recent years, there have been multiple calls for action to improve maternity services in the National Health Service (NHS) in England. Various formal independent reviews have been carried out in different regions of England with families of babies who had faced negative experiences in NHS care. These highlighted the need to improve the safety of maternity care in the NHS and put forward recommendations and actions needed to reduce the deaths of newborns and improve quality care. These reports included the Ockenden Report for Shrewsbury and Telford,^1^ the Thematic Review of incidents relating to Maternity Care at the Nottingham University Hospitals NHS Trust,^2^ and ‘Reading the Signals’ for East Kent.^3^ The main concerns raised in these reports included the disparities in outcomes for women of Black, Asian and Mixed ethnic groups and those who live in more deprived areas,^4^ which led to a call for urgent action in a letter published in the *BMJ*.^5^

Alongside this, there has been increasing importance placed on and interest in the involvement and engagement of service users in health and social care.^6^, ^7^ Involving service users is considered an important approach to improving the quality of health care because it ensures that the delivery of health care is adapted to the needs of the users, incorporating patient choice in the design of services and fostering a shared decision‐making model that embodies democratic principles and accountability.^8^, ^9^

Langley et al.^10^ identified co‐production as a method by which to integrate social factors into policy and practice. The diversity in evidence that can be incorporated into co‐production processes, and the relevance to patients, help connect evidence to policy and practice.^6^, ^10^ Reviews of the literature on co‐production have pointed to the lack of clear definitions related to what constitutes co‐production, with diverse and overlapping ideas over how this process is carried out.^6^, ^10^ This includes the interpretation of ‘co‐production’ as an umbrella term for the ‘co’‐ terminologies, such as ‘co‐design’, compared to its interpretation as a distinct term.^6^ The term ‘co‐design’ has also been highlighted to have unclear definitions,^7^ illustrating the unclear processes of service user involvement in health and social care. Rather than the necessity for clearly delineated definitions, these papers highlight the need for increased clarity over the values, principles and mechanisms that underpin the practices of co‐production and co‐design.^6^, ^7^, ^10^

Our paper sets out to build on the literature as we respond to this need through our evaluation of a co‐production project called Stories for Change, which sought to deliver a learning initiative based on co‐production, using co‐design methodology to involve service users in the redesign of maternity health services. Through our evaluation, the values and mechanisms that underpinned the co‐production approach are made explicit, and, consequently transparent and replicable.

### 1.1 Stories for Change background

In response to the inequity experienced in maternity care in the NHS, NHS England and NHS Improvement (NHSEI) developed a perinatal equity strategy.^4^ In October 2021, as part of this strategy, the South East region proposed a project with the objective of delivering a learning initiative on improvements in NHS maternity services based on co‐production with maternity service users, creating the Stories for Change project.^11^ The evaluation team adopted the term ‘maternity service users’ as this was the term chosen by NHSEI. The project included a co‐design group, and project organizers, who were made up of a service user representative, a subject matter expert on the Public Narrative approach, and a project administrator. The requirement for partaking in the co‐design group was having given birth in the NHS in the South East region within the last 2 years (2020–2022). All experiences were welcomed, mothers did not have to have faced explicit wrongdoing, but needed to have the desire to suggest improvements based on their experiences.

The Stories for Change project aimed to co‐produce solutions for the improvement of maternity services in the NHS. Based on the previous literature highlighting the complexity and variety in definitions, we have decided to make explicit the relevant definitions that guided this project's co‐production process. In this context, co‐production refers to the involvement of patients as partners in the improvement of the quality of health care, by being included in its design and production.^8^ Co‐design refers to a specific activity within co‐production, which entails their involvement in the process of designing a service, relating both to its functionality and to how it is experienced by service users.^8^

This project used the Double Diamond approach as a co‐design process. This approach embodies four steps to co‐design: discovering the service users' problems, defining the challenges in different ways, developing different answers to the problems and finally, delivering the solutions.^12^ This process incorporated the use of Public Narrative to develop the leadership resources of the co‐design group and to identify the service users' problems and develop solutions. This is a leadership practice put forward by Marshall Ganz that aims to use public stories to call others to action.^13^, ^14^, ^15^ The approach uses a method of storytelling based on values that draw on our emotional resources to galvanize others into creating change,^13^, ^15^ and the project aimed to use this approach to empower the leadership of the co‐design group to create change. The Public Narrative framework links a story of self to a story of us, which connects the speaker's personal values to the values of the audience, and leads to a story of now, which grounds these values into a call for action.^13^, ^14^ Another aspect of the co‐design process was the agreed norms and aims that were discussed and developed between the co‐design group and project organizers at every session, and included: active listening, camera on when possible, being present, respecting their diversity and everyone having a voice. The learning initiative centred the co‐design, content and delivery around developing the participants' skills, confidence and capability to use the Public Narrative approach to effectively lever change.

The project started in January 2022 and consisted of three co‐design group sessions, where the co‐design group members and project organizers would meet with the aim of developing the co‐design group members' stories. A Public Narrative skills session was held, where the co‐design group members developed their Public Narrative skills, and other maternity service users were invited to learn the methodology. The session created the opportunity for additional service users to join the project. The learning initiative culminated in the Learning Event in April 2022, where the co‐design group members presented their stories to NHS staff, to use their own lived experiences and voices to convey what could be improved in NHS maternity services. The co‐design group members decided who would be invited to this event, and some of the NHS staff invited were drawn from the same services as the co‐design group members, to directly express improvements they felt could be made.

## 2 METHODS

### 2.1 Study design

This study was designed as a rapid feedback evaluation with interviews as the main source of data.^16^ Using a rapid feedback evaluation allowed us to continually collect data and provide feedback within a limited timeframe.^17^ We undertook iterative processes of data collection and analysis, carrying out the two stages in parallel to share emerging findings and to inform subsequent data collection.^18^

The rapid evaluation was guided by the following questions:

1. What was the programme theory guiding the Stories for Change project? What were the expected outcomes?
2. What were the factors acting as barriers and facilitators to the implementation of co‐design and co‐production?
3. What was the perceived impact of the Learning Event on creating change?
4. How was the Public Narrative approach perceived by the different groups involved?
5. What recommendations did participants have for future similar projects?

### 2.2 Sampling and recruitment

Purposive sampling was used to recruit a sample of 16 participants (see Table 1 for the sampling strategy). We aimed to interview the different stakeholders involved in the co‐production process, this included the co‐design group members, the project organizers and NHS staff. We interviewed six co‐design group members, who were maternity service users learning the Public Narrative approach to present their stories to the NHS staff. Six were interviewed before the Learning Event, and five participated in a second interview after the Learning Event. We interviewed the three project organizers, whose role was to teach the Public Narrative approach, run the co‐design group sessions and guide the delivery of the Learning Event, with two acting as co‐facilitators and one as the project administrator. We also interviewed seven NHS staff who attended the Learning Event, to whom the stories were delivered and with whom the co‐design group members hoped to co‐produce solutions. The NHS staff recruited were all stakeholders in maternity services, which included managers, consultants, those involved in their Local Maternity and Neonatal System (LMNS), the project's co‐sponsors and Maternity Voices Partnership (MVP) co‐chairs. An MVP is an NHS working group made of stakeholders in maternity services, including service users, providers and commissioners that work together to improve maternity care.

**Table 1:** Sampling strategy for interviews.

	First interview	Second interview	Total interviews
Co‐design group members	6	5	11
Project organizers	3	0	3
NHS staff	0	7	7
	9	12	21

The project organizers worked with the evaluation team to recruit co‐design group members and NHS staff, by promoting the evaluation and inviting them to take part. Those who showed interest in participating were then contacted by one of the researchers (S. M.) via email. Participant information sheets and consent forms were shared, and interviews were arranged.

### 2.3 Data collection

In‐depth, semistructured interviews were conducted with the project organizers, co‐design group members and NHS staff over Zoom and Microsoft Teams from 14 February 2022 to 23 May 2022. The interviews were conducted by two researchers on the evaluation team working in parallel, using an interview topic guide, which was created based on the research questions guiding the study. The interviews were audio recorded, and interview data were entered into RREAL sheets, which helped to organize and summarize data in real‐time, based on key topics of interest.^19^ Organizing data in this way allowed the researchers to maintain consistency throughout data collection and identify the key findings of the study in a short amount of time.^16^

The interviews were conducted in two stages to capture the process of co‐designing and co‐producing the programme from beginning to end. The first stage was carried out with nine participants, who were co‐design group members and project organizers. The interviews captured their role in the project, their expectations, their experience of co‐design and co‐production and any barriers and facilitators to the co‐design and co‐production process. The second stage of interviews was carried out with 12 participants, which included co‐design group members interviewed in the first stage to capture their experience of the process over time, and NHS staff who attended the Learning Event. These captured their views of the Learning Event and what change they hoped the project would lead to. A total of 21 interviews were carried out. The interviews in both stages captured participants' views of the Public Narrative approach and recommendations for future similar projects.

Documentary analysis and observations were carried out to ensure triangulation using different data collection methods. This allowed us to include information that was not brought up in the interviews, address any knowledge gaps and analyse the intended objectives of the programme compared to what is happening in practice.^16^

### 2.4 Data analysis

We used an inductive‐deductive approach to data analysis, with the research questions guiding the analysis, whilst the evaluation team was sensitive to new themes arising from the interviews. Following rapid qualitative data analysis approaches, RREAL sheets were used to identify recurrent topics across study participants and enabled emerging findings to be shared in real‐time during the evaluation.^20^, ^21^

The RREAL sheet is a working document that enables the synthesis of data as data collection is ongoing.^21^ It allowed for the identification of gaps during data collection, and collaborative interpretation as regular team meetings were held to discuss findings.^21^ The RREAL sheets also helped identify when we reached data saturation, as it made clear when no new information was arising. The RREAL sheets were subsequently used to guide in‐depth analysis, after which the evaluation team discussed which quotes to use to illustrate the key findings. The manuscript was then shared with the project organizers, who reviewed the results and provided feedback.

Through using different RREAL sheets, research teams can make comparisons between different factors influencing the results, and thus for this evaluation, we used different RREAL sheets for each population and study stage (see Supporting Information: Appendix A for an example).^21^

## 3 RESULTS

The key findings related to the mechanisms and values underpinning the co‐production approach and recommendations for future projects and to create change. The mechanisms and values included: the collaborative and inclusive design of the project, power sharing, facilitation of an open and safe space, the use of stories and being called to action. Co‐design group members were also invited to share their lived experience perspective of taking part in this co‐production initiative.

### 3.1 Collaborative and inclusive design of the project

The co‐production approach was considered a process that was both collaborative and inclusive. Participants felt that collaboration was central to the co‐production process. Co‐design group members reflected on how the language used about collaboration was present in every part of the project.

The language that is used is all about collaborating, cooperating with one another, being part of this journey with us. (Co‐design group member)

Another component of the collaborative design of the project that was beneficial to the co‐design process was the diversity of skill sets amongst the project organizers. Participants believed that their mixed skillset created a balanced and dynamic group.

[Project organiser] is chairing the meeting, she's coming at it from a different way where she had to get the objectives covered and outcomes […] [project organiser] is there in the background providing additional support to all of us, but [project organiser] is on the other side providing emotional support […] quite a good dynamic of people. (Co‐design group member)

The project was thought to be inclusive due to the recruitment process. Participants agreed that the project had recruited a diverse group of mothers, mentioning factors including ethnicity, background and job roles. Remuneration enabled participation for some of those involved. Some mothers stated that it influenced them to get involved at the start, and participants expressed that it showed that their expertise and time were being appreciated. Participants had the option to get their childcare costs covered, and some decided to take that opportunity as they felt that it allowed them to be more present in the meetings. Remuneration also helped participants prioritize the project when they were busy.

I think it helps as well with commitment of time because everybody is so busy it helps to prioritise it. (Co‐design group member)

Project organizers acknowledged that people who the healthcare service had not worked with before would not have been aware of the project as recruitment was done through MVPs and social media accounts, which reached people with whom they were already connected. At the time of recruiting, they were also not aware that providing remuneration would interact with state benefits, which potentially led to the exclusion of participants from more deprived backgrounds.

We really want to make sure that we remunerate people for their time, which is great in practice but in reality […] the majority of the time if someone is in receipt of any state benefit they are unable to claim this money. (Project organizer)

Some co‐design group members voiced that systemic factors could have acted as potential barriers to certain populations participating. These included mothers who did not speak English well, who did not have adequate digital access to participate, and who did not have childcare options.

I wonder if actually some of the people who suffer the most from less effective care, less compassionate care, are people who don't speak very good English, people who don't have access to being able to join online groups, people who don't have the childcare support to be able to come join in those online groups. (Co‐design group member)

### 3.2 Power sharing

Co‐design group members expressed that power‐sharing was the norm. This meant that project organizers were genuinely collaborating with co‐design group members and not only using them as representation. This made co‐design group members feel that their expertise was being valued. They compared this project to others, where they felt that including the voice of service users was purely tokenistic.

This [group] is important because the power balance is right, whereas other groups and feedback requests and things like that you often feel like actually it's not real, you're going to say your thing but then the people running it are going to do their thing anyway, whereas this feels more like everyone's voice is important and they actually want to hear what needs to change rather than they've already decided. (Co‐design group member)

There were differing ideas in relation to the structure of the design. The project organizers had intentionally structured the project without an agenda to work with the co‐design group members to shape the content. At the beginning of the project, whilst some saw the nonhierarchical nature of the co‐design process as a facilitator to creating a collaborative space, others saw it as impractical. Participants acknowledged that this was due to shifting the power back to the service users, but some thought that it led to a lack of defined roles, and, therefore, practical steps, to guide the process. However, as the project progressed and participants reached the stage of planning for the Learning Event, all the co‐design group members agreed that the session added the practical component necessary to ground their stories and ideas.

The practicalities of the co‐design process are still a bit vague […] If everyone is brought to the same level, as in if the facilitators are really putting the power back to the group, that's a really nice idea but sometimes you need someone to guide it […] there needs to be some sort of concrete steps. (Co‐design group member)

### 3.3 Facilitation of an open and safe space

One of the most important mechanisms to the co‐production process was the open and safe space. This was a key theme that arose from interviews with the co‐design group members, which led them to feel empowered. The trusting relationship that was built in co‐design group sessions meant that they openly shared their ideas and stories. It enabled them to honestly address the changes that need to be made in the maternity system.

It's very open and free flowing, which makes me feel that there is a lot of trust there, we've been trusted to share what we think is best. (Co‐design group member)

NHS staff reflected on how the Learning Event provided a space for service users and service providers to communicate with each other directly, which prompted open conversations.

I think it worked very well because I think that in the subgroups, at least the group I was in, I felt that it really helped other professionals to open up and to connect with those people who had told the stories. (NHS staff)

The project organizers were considered important influences on the creation of this space. The co‐design group members were supported by the project organizers, who made the mothers feel that they could express sensitive issues such as racial bias and consent. Co‐design group members considered that the project organizers helped to facilitate conversation in a productive, yet not decisive way.

You can even express anything that is sensitive, like racial bias, like lack of consent […] but you forever feel supported while discussing it which is great. (Co‐design group member)

They were very good at prompting discussion without necessarily leading it. (Co‐design group member)

### 3.4 The use of stories

The use of stories was a key component of the Stories for Change project, and central to the co‐production approach of this project. The project organizers designed the project with the hope that through the service users constructing their stories using the Public Narrative approach, they would feel empowered and would influence the NHS staff to make a change.

To use the stories for change framework to build the skills, confidence and capability of current and recent service users to advocate on their own behalf for changes that matter for them. (Project organizer)

They also felt heard due to the common factor bringing them together in this project, which was that they all wanted improvements in the NHS maternity services. Telling their stories was deemed a silver lining of their birthing experiences, as speaking about it and producing change is something constructive that came from a bad experience.

I was thinking ‘my situation is quite different to everybody else’ […] but having ten faces on the screen and gradually get to realise that we are all in the same boat, it adds quite a lot of power. Everyone is gradually becoming quite empowered and that is going to have more impact going forward, for the people who want to listen and also for us. (Co‐design group member)

A key theme that emerged was the impact of emotion in the stories. Project organizers highlighted the importance of the use of emotion in stories to create the motion needed to lead to change and action. NHS staff found that there was an emotional resonance that other methods of sharing information do not have.

There's an emotional resonance there, that immediately you can connect with those people that you can't do by other means, you know a poster wouldn't have the same impact. (NHS staff)

### 3.5 Called to action

The NHS staff felt that the use of stories was a method that was conducive to learning and to creating change when staff were overworked.

The ‘stories’ bit in the title made it right […] to actually have an event that was focused in that with other key stakeholders there was really important. (NHS staff)

For NHS staff who attended the Learning Event, listening to the stories made them feel called to action. They perceived the stories to be attention‐grabbing, as this approach meant stories were told in a way that kept listeners alert. They acknowledged that listening to what could be improved in the services from maternity service users themselves was essential to giving the power back to the service users and making the appropriate changes.

That really important point of power sharing and giving that power back to the mothers and not thinking that you're the one that's sitting there with all the power. (NHS staff)

It made me feel that I am now in a position, a hot seat basically, to make a change with them. (NHS staff)

NHS staff and co‐design group members reflected on changes that started taking place as a result of the Learning Event. For example, NHS stakeholders stated that they would include information from the event in their revalidation reports, MVP co‐chairs recognized the importance of getting to know different cultures in their region, and maternity service users were involved in the development of training programmes in maternity services. The event also spurred a drive to use stories as a form of data, and participants stated that the Stories for Change project would lead to more similar events. On a personal level, NHS staff expressed how it would change the way that they interacted with their patients, as it opened their eyes to the importance of taking a more compassionate approach.

### 3.6 Lived experience perspective

**Figure 1:**
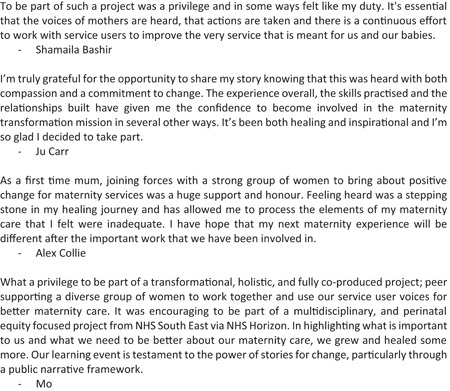
Lived experience commentary on the Stories for Change project on 10 August 2022.

Service users who took part in the Stories for Change project were also invited to contribute by sharing their lived experience perspective on the project. Figure 1 presents a commentary on the service users' experiences of taking part in this co‐production project.

### 3.7 Recommendations for future projects and to create change made by participants

During interviews, participants put forward recommendations for future similar projects targeted at specific stakeholders to ensure change in healthcare processes (Table 2). These include improving the recruitment and inclusion methods in the design process, factoring in diversity and types of remuneration offered to participants and improving the co‐design process by sharing their stories with each other earlier to facilitate improvement.

**Table 2:** Recommendations for future similar projects and to create change (made by participants).

Theme	Respondent group	Recommendations
Recruitment process	Project organizer	Long‐term networking is needed to connect with a variety of communities and established groups in these communities. Acknowledge the importance of building new relationships. Outreach workers (separate to community midwives) could follow up with people who register their pregnancy to give them the opportunity to be included in the project from the start. Ask what type of remuneration participants would want as state benefits may interact with remuneration and participants may not want to just receive money.
	Co‐design member	Local community outreach to include participants that would not hear about the event through social media or MVPs.
Inclusion in the co‐design group	Project organizer and co‐design member	Could include partners in future projects. Include more people from deprived backgrounds through improving recruitment and considering how to remunerate participants.
	Co‐design member	Could include someone that represents midwives as they would bring a different, more frontline perspective.
	NHS staff	Include mothers of the global majority who have English as their second language.
Co‐design process	Project organizer	Include daily themes (such as daily tips) in the WhatsApp group to create more connection.
	Co‐design member	At the start of the process, speak about which aspects of the project will be co‐designed and which will be co‐facilitated to mark the roles more clearly. Spend a whole day together to accelerate the process, either in person or online. Share their stories with each other earlier to better understand each other's experiences, what improvements they want to call for, and how to present them. Increase the number of sessions that they hold from the beginning to have more allocated time to create and share their stories and plan the event. Create an agenda before the meeting so they know in advance what would be discussed, and this would help them make a plan around what to do with their children during the event. Create a shared document to compile the notes that the co‐design members make reflecting on the process of the project.
Skills Session	Project organizer and co‐design member	Provide more time to set up and practice the Public Narrative approach.
	Co‐design member	Provide more time in breakout rooms. Make the Skills Session more of a workshop with role play and practice as the co‐design members had already learnt the skills. Invite participants earlier so that more people could attend.
Learning Event	Co‐design member	Encourage service user involvement through more social media use and a wider range of platforms (including Instagram). Hold more rehearsals. Implement a structure where everyone is prompted to talk in breakout rooms to encourage everyone to be present and to hear everyone's views. Encourage attendance from representatives from each co‐design member's local trust. Spend more time in the breakout rooms focussing on the pledges.
	NHS staff	Invite more consultants. Invite health visitors. Share more stories. Hold in person Learning Events.
In order to promote change: Accountability to commit to creating change	Co‐design member	Create a timeline and action plan to track the changes. Use social media to follow up on the decision‐makers' promises and keep the movement going. Hold a meeting every 3–6 months to get an update from the decision‐makers. Involve service users at the local level to ensure that they are listened to, that they can monitor the change, and keep the importance of the human experience on the service providers' minds. Hold a ‘you said, we did’ board for the professionals to show commitment to their actions.
	NHS staff	Hold an annual event to review progress. Constantly review progress through a health equity lens.
In order to promote change: Reaching a wider audience	NHS staff	Bring the project to every region and NHS hospital, or to individual provider trusts. Use eye‐catching advertisement to publicize the project. NHS employers should allow their staff time to attend and hear the stories. Learning Events should be held at board meetings as a mandatory event to reach people who do not want to listen to these stories.

Abbreviation: MVP, Maternity Voices Partnership.

### 3.8 Programme theory

**Figure 2:**
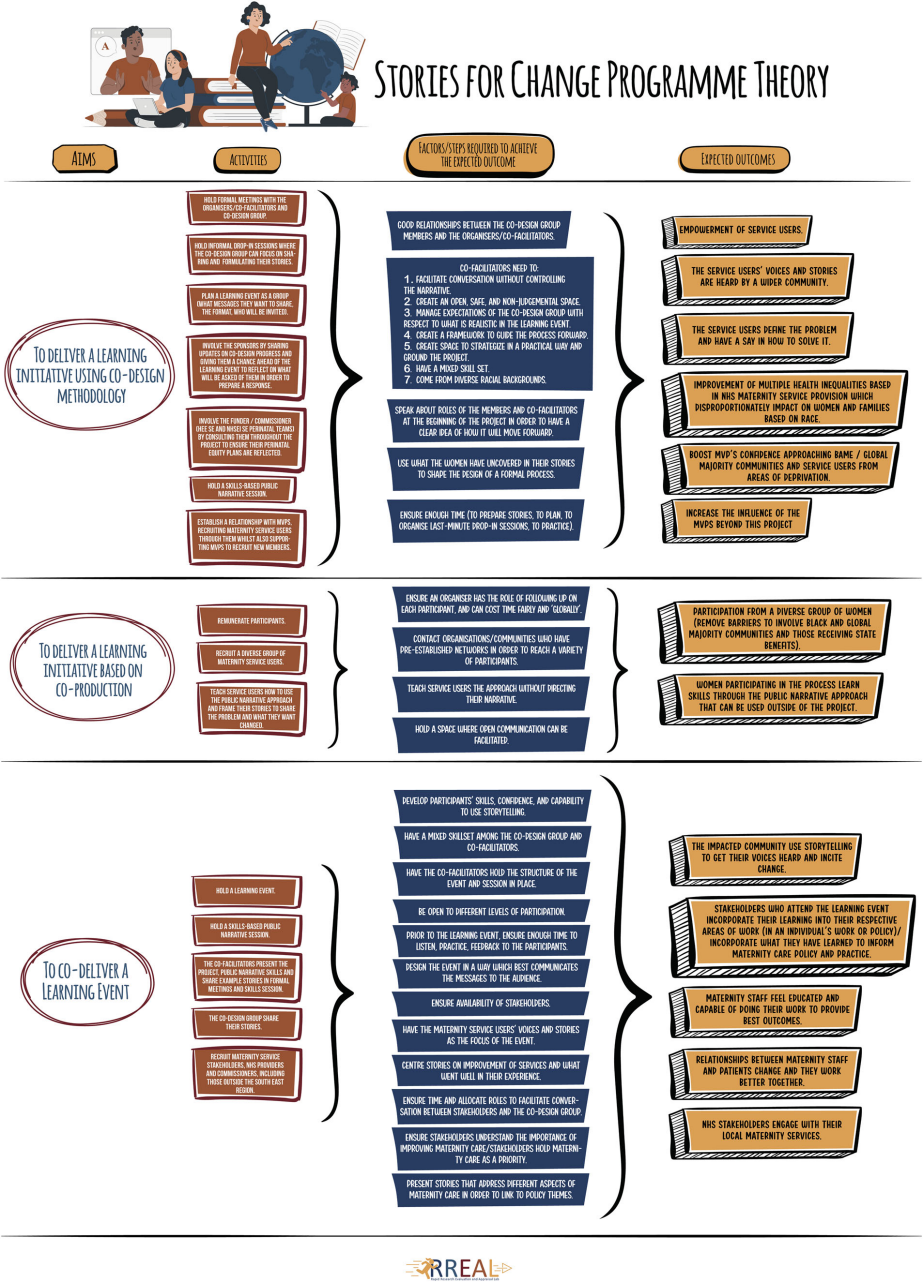
Stories for Change programme theory.

Based on interviews, observations and documentary analysis, we developed a programme theory for the project, which can be found in Figure 2. This programme theory serves as a useful framework on which to base future projects that wish to use the Stories for Change co‐production model.

## 4 DISCUSSION

Our study provided a deeper understanding of the mechanisms and values underlying a co‐production approach that aimed to involve service users in the redesign of maternity services. This study identified three broad factors in facilitating co‐production: creating an open and safe space to share ideas, learning how to tell stories using the Public Narrative approach and having service providers who play a key role in strengthening the health system listen to stories compelling them to action. These different factors provide insight into the influence of storytelling in creating meaningful change in healthcare, which could help inform future healthcare development processes.

The Public Narrative approach is a leadership practice that aims to express values through storytelling, and ultimately call people to action.^13^ This evaluation has shown that this method was conducive to contributing to co‐design in health care. Co‐design aims to value experiential knowledge,^22^ which is what the Public Narrative approach enables. The Double Diamond approach incorporates four steps, three of which are the discovery, defining and developing of the problems and solutions.^22^ Through storytelling, the co‐design group members were able to centre their values and experiences in the process of uncovering the problems and solutions in NHS maternity services. The broader context within which the Public Narrative was used fostered the creation of the service users as leaders, empowering them to use their stories and expertise to call for change. The findings highlighted that they were able to create an environment in which to openly uncover their stories and propose solutions, highlighting how the relationship between the approach and the values guiding the sessions enabled the use of the Double Diamond model to act as the co‐design process in this project.

Historically, the power balance between the professional and service user has been measured based on the product as opposed to the process, centring the importance around who the final decision‐maker is rather than the process of interaction between the different actors that leads to the production of solutions, as was the case with the Arnstein's iconic ladder of citizen participation.^23^, ^24^ The project's co‐production approach entailed co‐design methodology, which was shown to enable interaction between different actors, as maternity service users were incorporated into the process of redesigning services.^8^ Our study found that this co‐production approach is dependent on the nonhierarchical nature of the design process, which promoted power sharing. The Stories for Change project is an exemplar of the mixing of different forms of knowledge that represent an authentic co‐production experience.^25^

Our study found that having the space to share ideas allows for the recognition of everyone's expertise. Project organizers intentionally designed the project without content in order for the co‐design group members to have control over designing solutions. The facilitators and co‐design group members created a safe environment in which NHS staff could learn and have direct conversations with service users and consequently were open to changing their points of view and ways of working.

### 4.1 Findings in relation to other studies

Our results highlighted the importance of examining the process of producing solutions, as participants emphasized the importance of a collaborative and inclusive project and an open and safe space in empowering the service users and calling the NHS staff to create change. This aligns with previous studies identifying that successful co‐production was the consequence of having a ‘space to talk’ and a ‘space to change’, referring to the spaces that recognize everyone's expertise and allow for individuals to change their views.^25^ In our study, these spaces allowed for diverse forms of knowledge to interact on equitable grounds, which is considered important in dismantling the hierarchy of knowledge between professionals and service users in co‐production initiatives.^24^, ^25^, ^26^ The Public Narrative approach gives unique tools by which to challenge the knowledge hierarchy that maintains health professionals on a superior level, as it gives service users a technique by which to share their expertise of lived experience and connect with their audience.

Another study that supports our study was conducted by Abma et al.^27^ who promote a user involvement model called the Dialogue Model. Their study highlighted the importance of having a ‘safe environment’ as a foundation on which to develop the patients' voices, which consequently acknowledges power differentials and creates the space necessary for dialogue with professionals.^27^ Our study showed that in the context of maternity services, a safe environment is necessary to create a trusting relationship between stakeholders, as this fosters the honest sharing of stories and open dialogue between NHS staff and co‐design group members.

### 4.2 Strengths and limitations

To our knowledge, the present study is the first to use Public Narrative as a tool for service user engagement in health care. This is a strength as it makes explicit the mechanisms and values used to guide co‐design, which allows for future replication of this approach. Second, given the time limitations associated with developing healthcare policies, our rapid methodology allows for scientifically rigorous methods of ensuring that patients' voices are systematically identified and incorporated into healthcare policies in a timely manner.

The findings of this study should be considered in relation to its limitations. The data collection started when the project commenced, which meant that the first interviewees approached had little time to reflect. This was overcome by interviewing some interviewees in the second round, but not all took part in a second interview. Furthermore, due to the rapid nature of the evaluation, there was a short data collection period following the Learning Event, which meant that the impact of the project could not be properly evaluated. This also meant that we were unable to evaluate the whole Double Diamond model, due to the last step being the delivery of the solutions. Although the idea for the solutions has been delivered, we could not test and evaluate the impact of these solutions in maternity services. There may be biased perspectives on the positive impacts of the project, as the NHS staff interviewed maybe those who are more likely to attend such events, and who are more invested in creating change in maternity services than other NHS stakeholders.

## 5 CONCLUSION AND IMPLICATIONS FOR POLICY AND PRACTICE

Our study has shown a method by which co‐production can be done meaningfully with service users. We found that a co‐production approach that uses co‐design methodology with Public Narrative as a user involvement strategy empowers maternity service users and calls NHS staff to act and create change. Key to the co‐design process is the creation of an open and safe space that facilitates the dismantling of the hierarchy of knowledge between service user and professional, necessary to ensure that power is redistributed and that service users are genuinely part of designing solutions.

The results of our study carry with them some implications. First, this study suggests that creating impact through a co‐production approach can play an instrumental role in ensuring that patients' voices are accounted for in healthcare policy development processes. Second, the specific recommendations for incorporating stories in healthcare processes can also be applied to other co‐design projects that are unsure how to explicitly undergo co‐design. We recommend that future studies implement and evaluate the approach in other healthcare contexts.

## CONFLICT OF INTEREST STATEMENT

The authors declare no conflicts of interest.

## ETHICS STATEMENT

Ethics approval was obtained from the UCL Research Ethics Committee (REC) (6862/008).

## Supporting information

Supporting information.

## Data Availability

Audio recordings of the interviews analysed in this study cannot be shared publicly to protect the privacy and anonymity of participants. If you wish to obtain access to this data, please contact the UCL ethics committee at ethics@ucl.ac.uk and/or the corresponding author.
