# Supplementary material for: Stories for Change: The impact of Public Narrative on the co‐production process
Source: Health Expect. 2023 Jan 27;26(2):919–30. doi: 10.1111/hex.13718 (PMC10010083; doi:10.1111/hex.13718)
Supplement: Supplementary file 1 — Supporting information. [file HEX-26--s001.pdf]

RREAL SHEET FOR CO-DESIGN GROUP MEMBERS
STAGE ONE: PRE-LEARNING EVENT

- SFC= Storied for Change
- MVP= Maternity Voice Partnership

Categories	Main findings
How they found out about project	MVP<br>- Local MVP Facebook page (PE003, PE004); Her MVP co-chair told her about it (PE005); All the MVP chairs and co-chairs in the region received an email, and she is a co-chair (PE008)<br><br>Non-MVP related<br>- An advert on her pregnancy yoga Facebook page (PE007)<br>- Through project organiser, who thought it would be beneficial to have her input through her experiences with the maternity service before (PE009)
Reason for signing up to SFC project	- Hope to create change (PE003, PE004, PE005, PE007), feels a need for change in the NHS for Asian and all backgrounds (PE005)<br>- Using experience to create change whilst also using it as a cathartic process for herself (PE003)<br>- Awful experience, oldest child is 23 years old, went back to NHS for birth after 15 years and saddened that it hasn't changed (PE004)<br>- Name of the project "Stories for Change" really stood out to her (PE005)<br>- Passionate about birth and women; although she had a good birth on paper, she would have like the experience to be different in terms of communication and relationship with the midwife (PE007)<br>- Found the project interesting, excited about prospect of co-production, chance to make real change (PE008)<br>- Part of an initiative in Zambia that helps women overcome challenges; co-production aspect of the project appealed to her; to help out and give her opinion (PE009)
What they hoped to gain/ expectations for the project	- Help make change by using experience (PE004) and stories (PE005)<br>- After having started the project, also realises that the project has been beneficial on a personal level (PE004)<br>- NHS takes a long time anyways, so if this does lead to change, she doesn't expect it to take place for a long while (before her second child it will not happen); Voices may be heard, but that doesn't always lead to change (PE003)<br>- None as she didn't know what to expect, she knew she met the criteria for the project, and learnt about the objective of the project but not the road map (PE007)<br>- Wants the movement to go beyond the project; at the moment there is a large narrative (media) of scary maternity stories and doesn't want that to be the case, wants that to be the exception, wants the mothers to feel secure and cared for (PE008)
People involved in SFC and perception of inclusivity	Diversity<br>- Believe project is ethnically diverse (PE003, PE004, PE007, PE008) and in background (PE008)

	- Diverse group and inclusive of minorities (PE009)<br>- Most diverse group that she has ever been involved in (geographically, ethnically, and job roles- even have people who work for the NHS which she thought would get different treatment but turns out that that is not the case); at first thought she was really different as they all looked put together and confident; all had a baby in the last 2 years (PE004)<br>- Diversity relating to background, and their experience with the NHS because although they are all from the SE England, the approaches from the trusts are all very different and so someone 20 minutes down the road will have a very different experience, so diverse demographic-wise (PE007)<br>- Gave her the opportunity to speak to people who she wouldn't in her day-to-day role because her area is mainly white British (PE008)<br><br>Lack of Diversity<br>- Lack of inclusivity through systemic factors that are hard to address: (PE003) Not sure about socioeconomic diversity, May be a self-selection bias as they all speak good English, what about people who don't speak good English, or have the technical ability, or childcare support to join; How could they know about if they didn't follow their local MVP on Facebook
Suggestions to improve inclusivity	- Local community outreach, and then once you have people from these harder to reach communities, growing on that network (PE003)<br>- Have someone to represent midwives, they can create change and hold a different point of view; could include partners to see what their experience is although they don't go through the birthing trauma, they watch it which is trauma, important to have parents rather than just mothers' involvement, especially where safety was compromised (PE007)<br>- Suggests including someone to represent midwives to bring in that different perspective (PE007)<br>- Including dads and how they perceived it, especially in traumatic experiences they don't get involved because they aren't experiencing it, but watching it is traumatic- so could be that parents are involved rather than just mums (PE007)
Perception/feeling of involvement in co-design of SFC	- Her feelings change, sometimes she is excited, other times doubts whether people will actually listen to them. What brings them together is that nobody in the group was listened to about their experience, and now feels that together they can be listened to, and that their voices are being heard (PE004)
Role of non co-design maternity service users	- Sharing her story, her thoughts and opinions; kept up to date with the co-design group's plans and asked for her opinion (PE008)<br>- Participants, at first just learning; now has been invited to join the co-design group and is awaiting more info (PE009)

What co-designing SFC entails	- Have spoken about who to invite to the learning event, thought NHS leaders at first but have also decided that it is important to include other service users, choosing people who will make change (PE003, PE004)<br>- Flattening the hierarchy, which has pros and cons; Co-design seems tokenistic in the sense that someone is organising things behind the scenes etc., not in a bad way, just that practically you need someone to do this (PE003)<br>- No one knows exactly what to expect from the co-design, but realising that they are all in the same boat with respect to their experience has given them power, and through this empowerment they will have more impact (PE004)<br>- Making sure that they are all on the same page; they all have different experiences so they need to focus on what they can work on and on the specifics that they can identify what they want to change (PE007)<br>- Formal and informal sessions (PE003, PE004)<br>- Enjoy the drop-in sessions (PE004)<br>- Speak about themes, introducing the project, practice their stories in break out rooms and give each other feedback (PE003, PE004)<br>- They get to decide the different media they use, whether to use ppt etc. (PE003, PE004)<br>- Choosing how to frame their stories, and picking out the important messages that they want to share as a group, practising their storytelling and sharing why they feel they connect with the other stories, and picking out what needs to be addressed from the stories (PE004, PE005)<br>- Sharing stories in co-design group session (PE005, PE007); Leading breakout rooms in skills session; pitching idea to wider group who gave feedback and helped build confidence; telling story in skills session set her up for next co-design group (PE005)<br>- Praising each other; the co-design group put people forward with regards to who to invite to Learning Event (PE007)<br>- Informative process, sharing information after in your own time you can read, it has been very helpful and insightful (PE009)<br>- Cathartic process as they share experiences and relate to each other (PE008)
Barriers to co-designing	- The co-design aspect of the project is vague, find out as she goes along what it is about and what will happen next (PE003) ((PE004) also says that they find out as they go along but doesn't identify this as a barrier)<br>- Practicalities are vague; although power is given to the group, they need someone to drive the process forward in a practical way; lack of defined roles at the beginning; the co-design process seems idealistic; flattening the hierarchy: reality is that it is not practical if there is no one driving it forward, need a leader or leaders (PE003)<br>- Geographically dispersed group- meet every two weeks for 1.5 hours and spend the beginning recapping etc., don't spend a lot of time together (PE003)<br>- Doesn't identify any barriers, says that the project is tailored to the mothers and does not anticipate any barriers; is anxious about the triggers in presenting the story and not sure how to do it, but doesn't identify this as a barrier (PE004)

	- Project not being face to face, which would make it more coherent- online is more difficult as the session doesn't flow as easily, but acknowledges that with kids it is hard to go anywhere (PE005)<br>- Main barrier as lack of time: Haven't been able to fully share their stories with each other due to time pressure and has made collaboration harder as need more time; They haven't had a chance to share their stories with one another which means they can't say whether it's specific enough for the changes that they want to propose to the NHS leaders (PE007)
Facilitators to co-designing	- Feel supported by the facilitators, who recognise the challenges they have been through and telling their stories; Keeping the personal and private separate, have spoken about these boundaries from the beginning (PE003, PE005)<br>- Power is given back to the group, which is great; Flattening the hierarchy: recognises service user expertise, health care professionals/staff to not assume the power so levels them out; Informal 'drop-in' sessions are good as act as peer support (PE003)<br>- Power balance in this group is right, people who are running it aren't deciding what is happening, you feel you are being listened to and things are not pre-decided for you (PE004)<br>- Example stories provided by co-facilitators really help, provide lightbulb moment and not focussed on dwelling on the negative but on the bigger picture; not forced to share anything, made very comfortable; holistic focus of the project focus on improvements; feedback from the group; sharing stories- taking parts from another person's story that she found powerful (PE005)<br>- Expected more structure, telling her what to say, what was good and bad etc. but nothing like that; reality is that there is less structure which is good as they are not "ticking boxes", that it is very open and no one is telling them what to do; keeping it public and personal, distinguishing with the private; open-minded flow of discussions (PE005)<br>- Flexibility in hours together, Project Organiser has drop- in sessions, which can be late in the evening after the baby's bedtime; general approach by co-facilitators: aware that they are all mothers and that they have to feed, welcomed to take a break in the meetings, WhatsApp group makes the mothers feel supported, as they make time to write to each other and share, and they feel less alone (PE007)<br>- Sharing aspect: from MVP viewpoint realises through talking to other MVP of different trusts that they have similar problems, that the problems are widespread; from service user viewpoint listening to other stories creates solidarity- a mother reading out a poem and mother crying because they could relate, a cathartic process; open, non-judgemental, safe space to share her stories, never felt that she would be judged; but also as an MVP didn't feel ridiculed for sharing her ideas (PE008)<br>- An open, safe and non-judgemental space where they can share ideas; sharing stories can be hard so that atmosphere is conducive to telling these stories; she didn't feel uncomfortable voicing things from an MVP perspective either; it was speaking with the ethnically diverse SFC group that gave her confidence as an MVP to approach the BAME community as they gave her

	reassurance and gave her the idea to message the Imam of a mosque which previously she would have been worried to do (didn't know how to do it, would phrase it wrong); didn't want to speak to people of BAME background simply because of their background but they reassured her that they wouldn't be offended (PE008)<br>- Coming together and not feeling so isolated as everyone has experienced some element of what is going on as well; likes the breakup into groups and talk and then coming back to feedback into the wider group and no one is pressured to share what they don't feel comfortable sharing; feels that there was ample time which helped the group focus and formulate the network without being too much in each other's space (PE009)
Improvements to co-design process	- Would be useful to use some sessions to all share their stories because they don't share very much of their stories in the formal sessions, then they can get more familiar about what they are bringing and what their experience looks like, design for their stories may look different at the end because they haven't been constructing them collectively (PE007)<br>- Roles need to be more marked; Perhaps at the start of the process speak about what aspects of the project will be co-designed and what is facilitated (PE004)<br>- Too little time spent together= Spend a whole day in order to accelerate the process, whether it's in person or online. This will help keep up the pace (but acknowledges that emotions they deal with need processing and that a day could be heavy) (PE004)
What co-production entails	- All doing little bits which can create a big movement to galvanise change (PE008)<br>- Gives everyone a chance to participate; even husband who hasn't been visually present can tap into some of the things said and resonate with it (PE009)
Barriers to co-production process	- Goals for this project are too big to be achievable. A concern is that the other mothers are too aspirational wanting the UK to be the best place to give birth in the world, the goals too big to achieve and thinks they should strive for smaller goals in that direction. Hard to put names to what goals but e.g., reducing maternal and neonatal death by 10% (PE008)
Reflections on remuneration	- Did not expect remuneration, was not why she signed up (PE007, PE008)<br>- Generous, offer for childcare cost which has really helped as she can leave her daughter at the creche at the gym which is not only helpful because it prepares her for that when she goes to nursery, but also because it allows her to be present which is really positive (PE007)<br>- A confusing and not simple process, not because of the SFC side but because of pre-existing issues (PE008)
What co-delivering SFC entails	- Not sure what they will be doing to co-deliver, or what will be happening at the skills session (PE003, PE004)<br>- Not sure if the service users will co-deliver the sessions or if it will be led by facilitator (PE003)<br>- Some will be leading break out rooms and sharing their stories, others will be sharing the skills they have been learning, still in the process of discussing how everything will happen and what it will entail (PE004)

	- Delivering stories; the purpose is clear and there is an emphasis on power, explained how the way to use storytelling is the most precise way for them to be effective (short, precise) (PE007)
Collaboration in co-delivery	- More practical than co-designing, Co-design group has more opportunities as facilitators in this role, the service users are more the leaders, but in co-design collaboration it is difficult for someone to lead practically (PE003)<br>- The group has a mixed skillset which creates balance in the group- one facilitator creates structure etc. whilst the other one brings the lived experience which is more realistic and helpful, they are more inspiring together and they can learn from each other, although they share the same experience, they are all very different (PE004)<br>- Identifies co-delivery with the Learning event, and presenting story in the breakout room of skills session, but does not really relate co-delivery with the skills session (that is related to the co-designing) (PE005)
Barriers to co-delivery	- Nerves, but recognises that she will have to work with this (PE004)<br>- Last minute decision to lead the break out room in the skills session, would be better to be made clearer that they would be facilitating a session (PE007)
Facilitators to co-delivery	- They were asked if they wanted to lead break out rooms but were not pressured (PE005)
Reflections of skills session (SS)	- Agreed on WhatsApp before what each would be doing, there were different levels of involvement (PE007)<br>- If she had to pick only one meeting that they would have it would be the Skills Session; doesn't feel like she contributed much but she gained a lot (PE008)
Expectations for skills session	- Hopes to gain emotion to add to her SFC, at the moment she is feeling. Wants to add the personal and emotional as that is what instigates change. Feels vulnerable to be present (PE003)<br>- Didn't have many expectations as did not know much before (PE007)
What went well in skills session	- Feels that participants actively contributed, even the shyest of them all because they all felt comfortable and safe (PE005)<br>- Really well delivered, everyone was nervous, and they were all mums there and they felt comfortable; they felt a desire to come up with stories and felt more motivated because of the lack of pressure from the co-facilitators and group; co-facilitators took time to explain everything, anyone to ask questions and they gave answers (PE005)<br>- Gained confidence and ideas in how to perform better in MVP role to help reach the local community by learning better communication and verbalizing with the public; felt flung into the deep end in her role as an MVP and this felt like they were holding her hand and helping her; break-out rooms were managed very well, good mix of new people and experienced members, felt informed and supported; co-facilitators were good at stimulating conversation, asked questions and prompted responses but without leading them to a goal, gave them confidence to talk and share ideas (PE008)
Anything missing from skills session/ what could be improved	- Didn't have time to finish her whole story- more time for presentation of stories (PE005)<br>- Asking to lead break out rooms was a last-minute request; improvement would be to make it clear that co-design group members are responsible to facilitating a meeting (PE007)

	- Name was not appropriate as 'skills session' makes you think you'll come out with food for thought, for the other maternity service users attending it's empowering to learn but the co-design group had already learnt what they had done in the skills session; if they were to do another she would suggest doing a training and workshop where they feel they have learnt something new (co-design group members), although they have shared a project organiser's story in writing it would be better to do a role play scenario and practice what would happen for the final delivery (PE007)
Expectations for Learning Event	- Hopes to effect change through telling their stories (PE003, PE007), to go away with at least the hope that things might get better (PE003, PE008)<br>- For the Learning Event, expects the main focus to be on the messages they have to share, with the co-design group and others involved deciding how to go about it, and no one expected to do anything outside of their comfort zone (PE004)<br>- That there will be other mothers at the skills session and that the learning event will be delivered by the mothers, who will have the opportunity to tell the stories for change (PE003, PE004)<br>- Hoping not to cry, but expecting everyone to cry, will be the first time she'll hear some stories; it's a really big deal, speaking publicly on a platform like that and some people have never spoken in public; not about targeting the people in the audience (not "us" and "them", they are all human); expects people there to listen to what is said and hopes something is done about it-change; also expects for audience to ask questions to show that it means something to them because she also wants it acknowledged that this project is a lot of work and takes a lot of energy (PE005)<br>- That they will suggest things that people can go away with and implement into their practices (e.g. change guidelines, look at communication and recruitment of midwives); Hopes they have the confidence to share (there will be Senior NHS Leaders and people they do not know); excited to hear from people in the group as hasn't heard their stories yet (PE007)<br>- To be more of a listener than an active participant, she has more to gain than to share; excited to see who will be involved and expects influential and powerful people to attend and who have the power to push it forward (PE008)<br>- Expects a familiar, comfortable atmosphere that promotes active thinking and engagement; the maternity service providers give a lot to the service, and she sees it as a way that the service users can teach them, give ideas of change, a place for the service providers and users to connect (PE009)
Preparation for Learning Event	- Meetings before Co-design meeting #3 were more emotional (crying, sharing stories) and sharing stories but not about planning, the last meeting really helped plan and felt like a formalised strategy meeting that would help them move forward (PE008)
Other learning initiatives in the project (apart from Public Narrative approach)	- None that she knows of (PE003)<br>- She found the project overwhelming at first, so she used pens and paints to start art journaling and help her, which she shared with the group; personally, didn't find ppt as helpful, better to use a scrapbook to focus on what is important and use while storytelling; said other also used bullet point tables,

	some found talking and practising helpful whilst she found listening helpful to understand what engaged her (PE004)<br>- "Values, Emotions and Action" really stood out to her, and helped her story to focus on those aspects, understanding that it really is about taking the listener on a journey with you and to focus on the emotion in your story in order for them to feel the sense of urgency (PE005)<br>- Have had discussions about different ways to get their stories across including using diagrams and slide shows (PE007)
Perception of Public Narrative approach	- Has been really helpful for her role as MVP as she can tell the service users that this project is happening that is actively trying to improve service, gives her the confidence to speak to more people; she feels she can successfully access theme of perinatal equity, and the approach has raised her confidence (PE008)<br>- Useful and can incite change but not sure how far it can go; can access issues of perinatal equity and race through this approach; but questions how far it can go as different stories resonate with different people to tap into and therefore suggests that it should be kept on a smaller, regional scale in order to be more effective. Notes that this is partly because getting feedback from a lot of people can make it difficult to incorporate it all but mainly because it would be helpful to keep it according to different groups depending on their experiences, which is often linked to their postcodes (the "postcode lottery") (PE009)
Roles of co-facilitators and relationship with them	- Not therapists, but provide support them on their journey (PE003)<br>- Relationship with co-facilitators- from the beginning asked for contributions, realise they are working on something together and can express anything (racial bias, consent etc); they are active listeners and make her feel actively part of the group; language used focuses on collaboration and cooperation; feels involved throughout the whole process which is good because if she felt excluded she wouldn't share; feels trusted by the co-facilitators to decide what's best, the lack of pressure is ideal (PE0005)<br>- All co-facilitators bring something different: One is a service user whose presence is great as she provides emotional support and engages the participants, another makes the plan very clear, with the objectives and is very practical, and the other provides additional support which together creates a dynamic group (PE007)<br>- The co-facilitators are amazing- their energy, support and organisation, work well as a team and has been the best Zoom project she has been a part of, they are management without feeling like management, wouldn't have turned out this way without them, organising the movement (PE008)<br>- The mixture creates fruitful and diverse opportunities; good that they explain who they are and their role; different people from different departments bringing different perspectives; excited for the co-facilitators and co-design group to come together with their separate parts in the dress rehearsal and Learning Event (PE009)
Additional comments	- Knew little information before the first meeting, finds out as she goes along (PE003)<br>- [Project organiser] is inspiring (PE004)

	- Hard copies of slides to note down, can be sent to them in advance, always has to flip through to re-jog memory and make sure she is doing it right (PE005)<br>- For the skills session it would be helpful to know what the other maternity service users thought, and what it led them to do (for e.g. did they join their local MVPs?); the fact that the project is focussing on maternity health feels special as there is not always the time and funding for this (like obesity or the ageing population) and so she thinks that is really positive and is thankful for that (PE007)<br>- Honoured to be a part of the project; As an MVP, had a meeting with the director of midwifery because she spoke about the project; through the project she has felt more confident to approach BAME communities, before she felt it was daunting but now it validates why she is there, and can address them not as a tick box exercise but because she actively needs their involvement; so much that goes behind the scenes and honoured to be a part of the project (PE008)<br>- What went well- co-production was well handled, when there are a lot of people and it's hard to take in all their opinions- well structured, everyone is able to participate and engage further; use the strategies and information in her project helping women in rural areas in Zambia, where she can share NHS content and strategies to help them access healthcare (PE009)
